# The safety and efficacy of endobronchialvalve therapy in patients with advanced heterogeneous emphysema versus standard medical care: A meta-analysis

**DOI:** 10.1097/MD.0000000000012062

**Published:** 2018-08-21

**Authors:** Yu Du, Danyang She, Zhixin Liang, Wei Yang, Liangan Chen

**Affiliations:** a Department of Respiratory Medicine, Chinese People's Liberation Army General Hospital, Beijing; b Department of Respiratory Medicine, Affiliated Hospital of Hebei University, Baoding, Hebei, China.

**Keywords:** efficacy, endobronchial valves, heterogeneous emphysema, safety

## Abstract

**Background:**

Endobronchial valves had been utilized for many years to treat patients with advanced emphysema, despite unfavorable results. In this meta-analysis, we aimed to assess the efficacy and safety of the use of endobronchial valves in patients with heterogeneous advanced emphysema.

**Methods:**

We performed systematic database searches to identify clinical trials that met all our inclusion criteria. Direct-comparison and mixed-treatment-comparison (MTC) meta-analyses were conducted to estimate the mean difference or odds ratio of outcomes. Each outcome was analyzed with Review Manager 5 statistical software.

**Results:**

Eight prospective clinical trials assessing this therapy were retrieved, with a total of 744 patients. Outcomes, including the forced expiratory volume in 1 second (FEV_1_), 6-minute walk test (6MWT), and St. George's Respiratory Questionnaire (SGRQ), were analyzed, and the odds ratio of reported complications related to endobronchial valve therapy was calculated. Significant improvement in the mean difference of FEV_1_ (5.61 [4.42, 6.80]), 6MWT (25.75 [12.30, 39.20]), and SGRQ (−10.96 [−13.88, −8.05]) was observed after endobronchial valve treatment. Moreover, the rate of adverse events related to endobronchial valves was low.

**Conclusions:**

Endobronchial valve treatment offers benefits in terms of lung function and quality of life. Endobronchial valve treatment is feasible and safe for patients with advanced heterogeneous emphysema, especially those with no evidence of collateral ventilation.

## 1 Introduction

Chronic obstructive pulmonary disease (COPD) is a common preventable and treatable disease. Progressively persistent airflow limitation is the feature of COPD. The chronic inflammatory responses in the airways and lung to noxious particles or gases are enhanced in patients with COPD, which contribute to the overall disease severity. The mixture of small-airway disease (obstructive bronchiolitis) and parenchymal destruction (emphysema) contribute to the chronic airflow-limitation of COPD. COPD is a major public health problem and is projected to rank fifth worldwide in terms of burden of disease by 2020 and third in terms of mortality.^[1,2]^ COPD is receiving increasing attention from the medical community. But unfortunately, COPD remains relatively unknown or neglected by the public as well as by public health and government officials. Tobacco smoking is directly associated with the prevalence of COPD, yet in many countries, air pollution, including outdoor, occupational, and indoor sources such as the burning of wood and other biomass fuels, is a major risk factor for COPD.^[3,4]^

According to the National Emphysema Treatment Trial, lung volume reduction surgery (LVRS) can increase lung function, exercise capacity, quality of life, and survival in selected patients with emphysema.^[5,6]^ In addition, bronchoscopic techniques for the management of emphysema have evolved from the success of LVRS, which has been proven to alter the natural history of the disease. Bronchoscopic lung volume reduction (BLVR) with 1-way valves has been successfully attempted in both the laboratory and in selected clinical settings.^[7–16]^ It is projected that the most-affected emphysematous regions from ventilation can be excluded by endobronchial valves. As a result, if segmental or lobar resorption atelectasis can be induced, a physiological lung volume reduction can be expected. Therefore, patients with heterogeneous emphysema are ideal candidates for endobronchial valve therapy. Such a valve allows 1-way flow of secretions and air out of an occluded pulmonary segment during expiration but prevents distal flow during inspiration.^[7,8]^

A nickel-titanium (nitinol) self-expanding, tubular mesh that is covered with a silicone membrane supports the endobronchial valves. This equipment makes endobronchial valves to form a seal between the valve and the bronchial wall. The distal air and mucous are allowed to pass through the central duckbill, a 1-way exit. Before the valves begin to work, there are 2 works to do. First, to choose a suitable size of the valve, an endoscopic measurement gauge is used to size the bronchial diameter. Next, the loading catheter with the chosen valve is advanced to the target airway, and the valve is deployed via the working channel of a flexible bronchoscope. Furthermore, there are also emerging case report data on similar silicone valves that are inserted via rigid bronchoscopy^[9]^; these valves are easy to insert or remove.

The current data regarding comprehensive comparisons between endobronchial valve therapy versus standard medical care are not agreeable. The aim of our meta-analysis was to identify and analyze high-quality clinical trials on the efficacy and safety of endobronchial valve use in patients with heterogeneous advanced emphysema.

## 2 Methods

### 2.1 Search methods for identification of studies

We searched MEDLINE, Cochrane Library and ClinicalTrials.gov from 2003 to September 2013 using the following subject headings or keywords “Endobronchial valves,” “emphysema,” “patient,” “therapy,” “heterogeneous.” The search was restricted to English-language articles. The reference lists of review articles were also searched. If the outcomes from the original articles or the above clinical trials registers were insufficient, we contacted the authors or searched the US FDA web site for additional information.

Owing to the limited number of randomized controlled trials (RCTs), we did not include unpublished data. Trials were also excluded because of quality (design) or an insufficient data of patients.

### 2.2 Selection criteria

The inclusion criteria were as follows: original clinical trial; patients with heterogeneous emphysema; endobronchial valve treatment; trials that provided data regarding the percent change in forced expiratory volume in 1 second (FEV_1_) and distance on the 6-minute walk test (6MWT), rate of major complications, or St. George's Respiratory Questionnaire mean changes. We excluded trials if they included patients with asthma, involved non-predefined treatment arms, or were published only in protocols, abstracts, or non-English languages. The ethics committee of Chinese People's Liberation Army General Hospital had approved the study.

### 2.3 Data collection and analysis

Review Manager 5 statistical software was used to perform the analyses. The results are presented as the odds ratio or mean difference and 95% credible interval. Statistical significance was assessed by the *Z* test, and pooled data were considered to be statistically significant at *P* < .05

## 3 Results

### 3.1 Identification of eligible studies

The initial search returned 98 potentially relevant studies. After screening the abstracts, we excluded 81 that did not relate to endobronchial valve treatment of patients with advanced emphysema. After reading the full texts of the remaining articles, we excluded another 9 articles, as they were narrative articles that provided insufficient numerical results or were not clinical trials. Eight clinical trials,^[10–17]^ with a total of 744 patients, were ultimately included in this meta-analysis.

### 3.2 Study characteristics

Information regarding the 8 studies is listed in Table 1, all of which analyzed the change in FEV_1_ and the safety of endobronchial valve therapy. Seven of the 8 studies analyzed the change in 6MWT, and 5 analyzed the change in St. George's Respiratory Questionnaire (SGRQ).

**Table 1:**
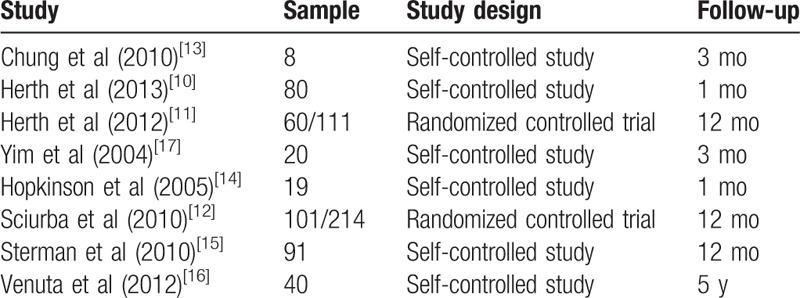
Information of included studies.

### 3.3 Effect of endobronchial valves on FEV1

**Figure 1:**
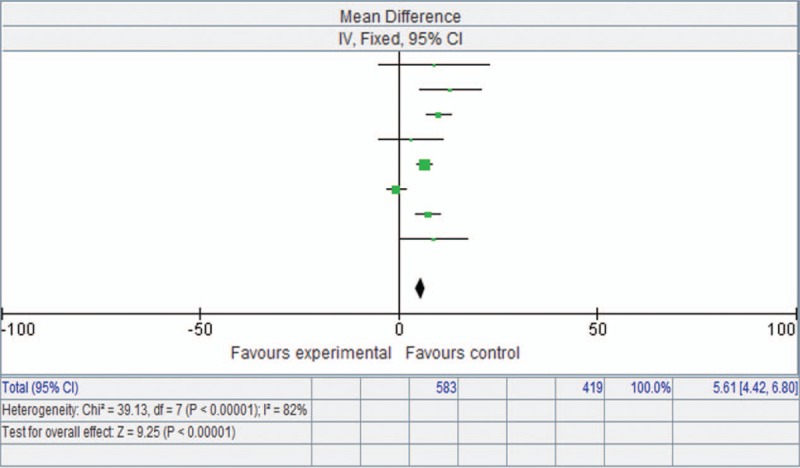
Forest plot showing forced expiratory volume in 1 secondbased on a fixed-effects model.

The meta-analysis of the 8 studies showed significant improvement in FEV_1_ with endobronchial valve therapy. The mean difference was 5.61% (95% confidence interval [CI] 4.42–6.80, *P* < .00001) (Fig. 1).

### 3.4 Effect of endobronchial valves on 6MWT

**Figure 2:**
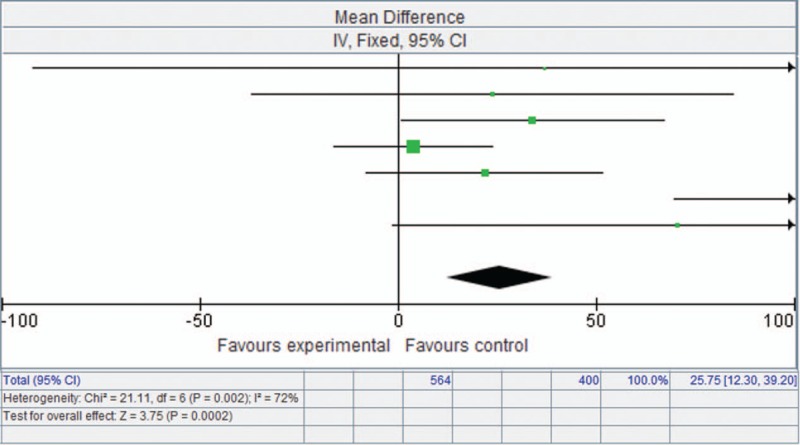
Forest plot showing 6-minute walk test based on a fixed-effects model.

According to meta-analysis of the eight studies, 6MWT also improved significantly with endobronchial valve therapy. The mean difference for this variable was 25.75 m (95% CI 12.30–39.20, *P* = .0002) (Fig. 2).

### 3.5 Effect of endobronchial valves on SGRQ

**Figure 3:**
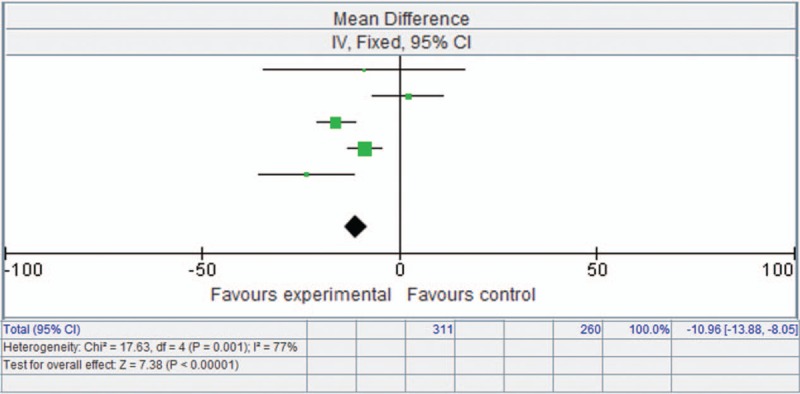
Forest plot showing St. George's Respiratory Questionnaire based on a fixed-effects model.

Similarly, SGRQ was significantly enhanced with endobronchial valve therapy, with a mean difference of −10.96 points (95% CI [−13.88, −8.05], *P* < .00001) (Fig. 3).

### 3.6 Safety of endobronchial valve therapy

Six of the 8 studies were self-controlled studies, and information regarding rates of complications was insufficient. We analyzed all reported adverse events in the studies, and 68 of 583 (11.66%) patients who underwent the endobronchial valve procedure experienced adverse events. However, no patient died as a result of the procedure.

## 4 Discussion

COPD and emphysema will become anincreasingly serious social and medical problem in the future. Indeed, the World Health Organization has suggested that emphysema will likely become the third-leading cause of death, along with cigarette smoking.^[10,18]^ Of concern, conservative medical therapies cannot provide satisfactory long-term therapeutic efficacy.^[11,19]^ Regardless, the mortality and efficacy of LVRS remain controversial. As an alternative to this approach, endobronchial valve therapy has been evaluated in many clinical trials, although the safety and efficacy of endobronchial valves were not satisfactory. Therefore, we deemed it essential to synthesize the clinical trials published to date in an effort to evaluate the safety and efficacy of endobronchial valves by meta-analysis.

This study included eight clinical trials. The pooled data showed that endobronchial valve therapy can improve lung function, exercise capacity, and quality of life, with a low rate of adverse events.

FEV_1_ of the endobronchial valve group was considerably increased, with a mean difference of 5.61 (4.42, 6.80), after the procedure, in accordance with the results of previous studies. Furthermore, lung function indices tended to become stable from 1 year after the procedure,^[11,15,16]^ a finding that needs to be evaluated by further RCTs.

In addition, a satisfactory effect of endobronchial valve treatment on 6MWT was also observed, with a mean difference of 25.75 (12.30, 39.20), consistent with outcomes in previous studies. Venuta et al^[16]^ even suggested that exercise testing improved continuously after the procedure.

In contrast, Sterman et al^[15]^ reported that neither FEV_1_nor 6MWT showed significant improvement.

With respect to SGRQ, the mean difference was −10.96 (−13.88, −8.05), suggesting that endobronchial valve treatment can also substantially improve quality of life, in accordance with outcomes in previous studies.^[10–17]^

Nonetheless, not all patients with advanced heterogeneous emphysema will improve with endobronchial valve treatment. Wan et al^[5]^ conducted an international multicenter cohort study in which 98 patients suitable for LVRS underwent endobronchial valve treatment, with improvements in FEV_1_ and 6MWT reported. The greatest magnitude of benefit was found in unilaterally treated patients with lobar exclusion and patients with lower baseline FEV_1_. In addition, Herth et al^[10,11]^ found that superior clinical results correlated with computed tomography (CT) findings suggestive of complete fissures and successful lobar occlusion. The Chartis pulmonary assessment system can be a useful tool to aid clinicians in planning endobronchial valve treatment.^[10]^

Overall, the safety of endobronchial valve treatmentremains controversial, yet the pooled data of our meta-analysis suggest that the rate of adverse events of the procedure is low. One of the 2 RCTs^[12]^ included in this study reported a rate of complications of 6.1% and 1.2% in the endobronchial valve treatment and control groups, respectively (*P* = .08), by 6 months; however, at follow-up from 6 to 12 months, the rate of complications among patients in the former (4.7%) was similar to that in the latter (4.6%). This resulted in an overall rate at 12 months of 10.3% in the endobronchial valve treatment group and 4.6% in the control group (*P* = .17). The other RCT^[11]^ reported the rates of valve expectoration, aspiration or migration of 7.2% (8/111) at 90 days, 0.9% (1/111) between 98 and 194 days, 3.6% (4/111) between 195 and 284 days, and 0.90% (1/111) between 285 and 386 days in the endobronchial valve treatment group. Lastly, Wan et al^[5]^ reported that 8 patients (8.2%) developed serious complications, including 1 death (1%) in the first 90 days.

Despite the different assessment criteria of the included clinical trials, all of the studies concluded that endobronchial valve treatment can improve lung function, exercise testing, and quality of life. There are many mechanisms for improvement with endobronchial valve treatment. The original hypothesis was that blocking an airway would cause lobar atelectasis to emulate lung volume reduction.^[20]^ The second mechanism is dynamic hyperinflation reduction.^[14]^ The third mechanism involves interlobar shift of ventilation from the treated upper lobe to the untreated lung zones identified by serial quantitative CT, as reported in 2008.^[21]^

There are a number of limitations to this meta-analysis. Unpublished trials and any other works were not included, and the omission of these potentially related studies may have influenced our conclusions. Second, the clinical trials included in our study were not all RCTs, and we would be more confident in our conclusion if more RCTs were available. Furthermore, the follow-up period in the clinical trials varied widely, and not all the trials compared all outcomes. Consequently, further high-quality RCTs are required to evaluate the long-term efficiency and safety of endobronchial-valve therapy in patients with advanced heterogeneous emphysema. The effect of endobronchial valves on the prognosis of emphysema still needs to be determined.

## 5 Conclusion

Despite the potential adverse events related to the procedure or the implants, endobronchial valve treatment results in more benefits in terms of survival, lung function, quality of life, and exercise capacity. As a result, endobronchial valve treatment should be recommended for suitable patients whose CT scan shows advanced heterogeneous emphysema. We prefer the Chart is pulmonary assessment system before therapy for selecting suitable participants and for predicting the success of the therapy.

## Acknowledgments

The authors acknowledge Feng Rui-e, Peking Union Medical College Hospital, for the assistance with the histopathological pictures. The authors also acknowledge the radiology department of Chinese People's Liberation Army General Hospital for the assistance with the Radiography images.

## Author contributions

**Data curation:** Yu Du.

**Formal analysis:** Yu Du.

**Investigation:** Yu Du, Danyang She, Zhixin Liang, Wei Yang.

**Methodology:** Danyang She, Liangan Chen.

**Project administration:** Liangan Chen.

**Resources:** Zhixin Liang.

**Software:** Danyang She.

**Supervision:** Liangan Chen.

**Validation:** Wei Yang.

**Writing – original draft:** Yu Du.

**Writing – review & editing:** Wei Yang.
